# Supplementary material for: How does handwashing behaviour change in response to a cholera outbreak? A qualitative case study in the Democratic Republic of the Congo
Source: PLoS One. 2022 Apr 12;17(4):e0266849. doi: 10.1371/journal.pone.0266849 (PMC9004767; doi:10.1371/journal.pone.0266849)
Supplement: S1 Table — (DOCX) [file pone.0266849.s001.docx]

Supplementary Materials – 1

Table 1: Handwashing determinant definitions adapted from on the BCD checklist of determinants (1, 2) and accompanied by method selections.

| **Behavioural determinants defined by the BCD framework** | | **Definitions of each determinant adapted to handwashing** | **Methods contributing to understanding this determinant** |
| --- | --- | --- | --- |
| **Brain (Cognitive factors)** | Executive Brain | The extent to which knowledge of handwashing behaviour and its benefits affects handwashing intentions and plans, and eventually performance of the behaviour. | - Risk scaling - Free-listing and ranking problems |
|  | Motivated Brain | The goal-related drivers of behaviour. Motives for handwashing can include disgust (the desire to avoid cues to sources of infection), affiliation (the desire to fit in with others) and nurture (the desire to care for your child) | - ‘How do you feel?’ Activity |
|  | Reactive Brain | The extent to which handwashing can be automatically triggered based on past experience and repetition. | - Observations - Handwashing Demonstrations |
|  | Discounts | The perceived time, effort, cost and benefit of washing hands with soap as compared to other courses of action. | - Problem free-listing and categorisation - Free-listing and ranking problems |
| **Body (Individual characteristics)** | Characteristics | Socio-demographic characteristics that may affect handwashing including gender, wealth, age, education, employment and personality. | - Socio-demographic survey |
|  | Capabilities | Whether an individual has the skills required to wash their hands with soap. Whether an individual perceives themselves to be able and willing to actually wash their hands at the times required. | - Observations - Handwashing Demonstrations - Problem free-listing and categorisation - Free-listing and ranking problems - Trials of improved practice |
| **Behaviour settings** | Stage | The design and set up of the specific physical spaces where handwashing behaviour takes place. | - Observations - Handwashing Demonstrations |
|  | Infrastructure | Durable infrastructure associated with handwashing such as water supply systems, sanitation or kitchen facilities and handwashing facilities. | - Designing the ideal handwashing facility - Water prioritisation - Handwashing Demonstrations - Free-listing and ranking problems |
|  | Props | The value, characteristics, usability, ownership and accessibility of soap and other objects used for handwashing. | - Soap Attributes - Handwashing Demonstrations - Free-listing and ranking problems |
|  | Roles | The ways in which an individual’s role, identity or responsibilities influence their handwashing practices. | - Identity Questionnaire - Personal Histories |
|  | Routine | The sequence of behaviours regularly performed in association with handwashing. | - Routine scripting - Personal Histories |
|  | Norms | The extent to which an individual’s handwashing practice is influenced by their perception of normative setting-specific rules. This includes an individual’s perception of whether handwashing is commonly practiced in their community (descriptive norm); whether handwashing is part of their role and their normal behaviour (personal norm); whether handwashing is socially approved of (injunctive norm); and whether handwashing is practiced by their ‘valued others’ (subjective norm). | - 100 people |
| **Broader Environment**  **External context** | Physical and biological environment | Physical environment includes the factors in the natural or built environment including climate and geography. Biological environment includes the factors associated with an individual’s interaction within their biological environment including disease vectors. | - Observations - Problem free-listing and categorisation - Free-listing and ranking problems |
|  | Social Environment | The structure of an individual’s social environment, including how they interact with it and perceive themselves within it. | - Social Network Diagrams - Personal Histories |
|  | Political and historical context | The historical and cultural events that have shaped current perceptions and practices of handwashing. The extent to which handwashing-related policies or local and national leadership on handwashing issues, shape handwashing perceptions and practices at the individual level. | - Problem free-listing and categorisation - Observations - Personal Histories |

# References

1. Aunger R, Curtis V. BCD Checklist. Behaviour Centred Design Resources Website: LSHTM; 2019.

2. White S, Thorseth AH, Dreibelbis R, Curtis V. The determinants of handwashing behaviour in domestic settings: An integrative systematic review. International Journal of Hygiene and Environmental Health. 2020;227:113512.
