# Supplementary material for: How does handwashing behaviour change in response to a cholera outbreak? A qualitative case study in the Democratic Republic of the Congo
Source: PLoS One. 2022 Apr 12;17(4):e0266849. doi: 10.1371/journal.pone.0266849 (PMC9004767; doi:10.1371/journal.pone.0266849)
Supplement: S2 Table — (DOCX) [file pone.0266849.s002.docx]

Supplementary Materials – 2

Table 1: Description and sample size for all methods done at a household or individual level.

| **Method** | **Description** | **Purpose** | **Origins and prior use** | **Sample** |
| --- | --- | --- | --- | --- |
| Observations | Unstructured observations at the household level. 3 hours in duration, beginning at 8am. Observers wrote down all actions that they observed and the time they took place at. Observation included the actions of all household members. | Unstructured observation provides rich, contextual detail about how handwashing behaviour fits within broader daily routines and the community and household environment. This was particularly relevant in crisis contexts where populations may still be adjusting to new or different physical environments. Observation can also highlight unforeseen barriers to desirable handwashing behaviour. | Observation is commonly used both to understand and monitor handwashing behaviour (1-3). While it has limitations, it is recognised as the gold-standard for handwashing measurement. | 16 |
| Trials of Improved Practices | Participant households were interviewed three times over the course of two weeks. During the first visit households were given soap and informed about the critical times for handwashing. They were then asked to encourage all family members to wash their hands at these times over the next two weeks. | To understand whether populations in this setting were able to make changes to their social and physical environments to facilitate handwashing practice | TIPs have been used in several previous hygiene studies and aim to understand feasibility and acceptability of improving the practice of target behaviours (24, 25). | 6 |
| Routine Scripting | Ask the participant what they did yesterday from the moment they got up in the morning right through to when they go to bed at night. Ask them to describe their routine step-by-step. Draw/write each activity on a separate piece of card. Lay the cards out in order. Explore parts of their routine that are of interest to handwashing and which parts of their day have changed since the crisis. Ask the participant what are the best, worst, most boring and most rewarding moments of their day. | It is designed to document the order of actions in people’s day-to-day routines. It explores whether the crisis disrupted or changed these routines and whether this has compromised people’s ability to practice handwashing with soap. | Similar methods have previously been used to explore handwashing (4) and other behaviours (5). | 9 |
| Personal Histories | Participants are given a blank piece of paper and asked to draw a picture of themselves before the crisis on the left hand side and a picture of themselves currently on the right hand side. Draw a line between the two images to represent the journey the individuals went on between these time points. Ask the participant to help you draw on some of the key milestones that happened in their lives during this time (whatever they are comfortable with sharing). Ask them to describe how they felt during these key time points and how their routines and behaviours changed. At the end ask about whether their handwashing behaviour changed. | This method was designed to generate a broad understanding of the context within which handwashing is situated and provides insights into the participant’s emic beliefs and culturally constructed understanding handwashing behaviour, crisis and disease.  Cultural beliefs and attitudes about a wide range of things may influence handwashing practice. In some humanitarian emergencies traditional beliefs and rumours may become a more dominant influence on people’s behaviour as they search to make sense of the new circumstances they find themselves in. | Narrative interviewing is particularly common in sociology (6-8) yet infrequently used within the WASH sector. | 8 |
| Free-listing and ranking problems | This activity uses the emergent category from the FGD discussion (in this case ‘hygiene problems’). Introduce the category to the participant and ask them to free-list any hygiene related problems they are currently facing. Draw/write each on a separate piece of card. Once all problems are listed ask the participant to rank them in order of priority. If not mentioned ask if handwashing with soap is problem and get them to insert this into the ranked order. | Free listing is particularly useful for mapping emic understandings of social domains, while ranking can provide insight into how these are structured and classified (9). Here we used it to understand the relative importance of handwashing in relation to other hygiene concerns. Through this method we were interested in exploring whether changes in risk perception in the wake of a crisis or an increased number of other competing priorities may affect handwashing prioritisation in relation to other hygiene activities. | Free-listing and ranking are both tools which are broadly used in qualitative research (7). Both have been used widely in WASH related research (10, 11) and in research among crisis affected populations (12, 13). | 9 |
| Handwashing Demonstrations | The researcher asks the participants to show them how they normally wash their hands. With permission the researcher videos the handwashing process. The researcher should pay attention to the behavioural setting, things that enable or create barriers to the behaviour and moments of uncertainty or hesitation that indicate that this may not be part of normal routines. | This method generates quick insights into the barriers and enabling factors related handwashing behaviour. Handwashing Demonstrations are also particularly useful for identifying whether the behaviour is one that is familiar or performed irregularly. | This method has been used in some other WASH studies (10) | 10 |
| Social Network Diagrams | Explain that you would like to understand more about social relationships in this place and in the place where they came from (prior to the crisis). Draw three concentric circles on a piece of A3 paper. The inner circle is for people who the participant meets daily. The middle circle is for people they meet weekly. The outer circle is for people the meet monthly or less frequently. Divide all the circles into sections, one for acquaintances, one for friends and one for family. Map the participant’s relationships prior to the crisis and ask how they are different now. Discuss what has changed, and which individuals are likely to influence their behaviour the most. | Social networks are likely to be important for establishing norms in relation to handwashing and for encouraging adherence. However, in humanitarian emergencies social networks may be disrupted either by displacement or by a reduction of social interactions as a consequence of a disease outbreak. | Social network analysis is a widely-used approach in sociology. It is often used to understand the diffusion of ideas or innovations and explore patterns of influence within social relationships (14-19). We did not find any examples of it being used within the WASH Sector. | 6 |
| 100 people | Participants are asked to imagine a sample of 100 people in their community (this is represented through 100 counters) and then asked to make predictions about their beliefs and behaviour.  Questions include how many people would tell us (the researchers) that they wash their hands, how many do they think actually wash their hands, how many would judge them negatively if they saw a person not washing their hands with soap, how many believe handwashing is the right thing to do and how many would say handwashing is easy.  Twenty counters are then selected and the participant is asked to imagine that these are their closest family and friends. The questions above are repeated for this group. | This method is designed to understand so that descriptive and injunctive norms about handwashing.  In stable settings norms and perceptions of social judgement influence handwashing behaviour. Humanitarian emergencies are likely to cause short term or long-term shifts in norms, but to date there has been little research to understand this. | While there are lots of methods for assessing norms, this method was chosen because it is easy to use with audiences with limited literacy (20). | 6 |
| Identity questionnaire | Participants are asked about their characteristics (e.g. abilities, possessions, career), personal identity (e.g. values, beliefs, feelings), social identity, (e.g. how they think others perceive them) collective identity (e.g. religion, nationality, culture) and the relational identity (e.g. their roles as friend, parent). They uses a three-point colour coded Likert scale to describe how important each item was to their sense of self. | The purpose of this method is to understand how participants perceive themselves and how they may juggle their different identities – each of which may influence handwashing in different ways.  A person’s perception of their roles and identities may influence handwashing in a range of ways. For example, major life changes (e.g. pregnancy) have also been found to have an effect on handwashing (4). Exposure to traumatic events change people’s perception of themselves and affect behaviour (21, 22). This method aimed to disentangle which aspects of identity had changed and how this may affect handwashing behaviour. | This method uses an adapted version of the Aspects of Identity Questionnaire (23). To the best of our knowledge that has not been previously used within the WASH or humanitarian sectors. | 8 |
| Water prioritisation | This method was only done in the villages where water was scarce and had to be collected in jerry cans. Ask the participant to show you the containers they use for water collection. Ask how much water they collect per day. If for example, they say 5 containers, use 5 plastic cups to represent this for the activity. Using the cups, get the participant to demonstrate how much water is normally used for different household activities. Note whether handwashing is mentioned as a use of water. Ask the participants whether they ever collect more water than normal. When does this happen? Add cups as necessary and again ask how they divide up the water for different purposes when they have more available. Ask the participant whether they ever collect less water than normal. When does this happen? Remove cups as necessary and again ask how they divide up the water for different purposes when they have less available. | It is designed to understand how patterns of water use within the household may respond to fluctuations in water availability and how this may influence handwashing behaviour. | This method is based on previous formative research on WASH behaviours and trachoma (29) | 14 |

# References

1. Ram PK, Halder AK, Granger SP, Jones T, Hall P, Hitchcock D, et al. Is Structured Observation a Valid Technique to Measure Handwashing Behavior? Use of Acceleration Sensors Embedded in Soap to Assess Reactivity to Structured Observation. The American journal of tropical medicine and hygiene. 2010;83(5):1070-6.

2. Ram P. Practical Guidance for Measuring Handwashing Behavior. WSP website: Water and Sanitation Program 2010.

3. Biran A, Rabie T, Schmidt W, Juvekar S, Hirve S, Curtis V. Comparing the performance of indicators of hand-washing practices in rural Indian households. Tropical medicine & international health : TM & IH. 2008;13(2):278-85.

4. Greenland K, Iradati E, Ati A, Maskoen YY, Aunger R. The context and practice of handwashing among new mothers in Serang, Indonesia: a formative research study. BMC public health. 2013;13:830-.

5. Aunger R. Tooth brushing as routine behaviour. International Dental Journal. 2007;57(S5):364-76.

6. Mishler EG. Models of Narrative Analysis: A Typology. Journal of Narrative and Life History. 1995;5(2):87-123.

7. Beebe J. Rapid assessment process: An introduction: AltaMira Press; 2001.

8. Bates JA. Use of narrative interviewing in everyday information behavior research. Library & Information Science Research. 2004;26(1):15-28.

9. Borgatti SP. Elicitation Techniques for Cultural Domain Analysis. . In: Schensul J, editor. Ethnographer's Toolkit. Newbury Park: Sage; 1998.

10. White S, Kuper H, Itimu-Phiri A, Holm R, Biran A. A Qualitative Study of Barriers to Accessing Water, Sanitation and Hygiene for Disabled People in Malawi. PLoS ONE. 2016;11(5):e0155043.

11. Domènech L, Heijnen H, Saurí D. Rainwater harvesting for human consumption and livelihood improvement in rural Nepal: benefits and risks. Water and Environment Journal. 2012;26(4):465-72.

12. Palmer CA. Rapid appraisal of needs in reproductive health care in southern Sudan: qualitative study. BMJ. 1999;319(7212):743-8.

13. Betancourt TS, Speelman L, Onyango G, Bolton P. A Qualitative Study of Mental Health Problems among Children Displaced by War in Northern Uganda. Transcultural Psychiatry. 2009;46(2):238-56.

14. Centola D. The Spread of Behavior in an Online Social Network Experiment. Science. 2010;329(5996):1194-7.

15. Bond RM, Fariss CJ, Jones JJ, Kramer ADI, Marlow C, Settle JE, et al. A 61-million-person experiment in social influence and political mobilization. Nature. 2012;489(7415):295-8.

16. Abrahamson E, Rosenkopf L. Social Network Effects on the Extent of Innovation Diffusion: A Computer Simulation. Organization Science. 1997;8(3):289-309.

17. Kempe D, Kleinberg J, Tardos É. Influential Nodes in a Diffusion Model for Social Networks. In: Caires L, Italiano GF, Monteiro L, Palamidessi C, Yung M, editors. Automata, Languages and Programming: 32nd International Colloquium, ICALP 2005, Lisbon, Portugal, July 11-15, 2005 Proceedings. Berlin, Heidelberg: Springer Berlin Heidelberg; 2005. p. 1127-38.

18. Scott J. Social network analysis: Sage; 2012.

19. Borgatti SP, Mehra A, Brass DJ, Labianca G. Network analysis in the social sciences. science. 2009;323(5916):892-5.

20. Bicchieri C, Lindemans JW, Jiang T. A structured approach to a diagnostic of collective practices. Frontiers in Psychology. 2014;5:1418.

21. Ullrich PM, Lutgendorf SK, Stapleton JT, Horowitz M. Self regard and concealment of homosexuality as predictors of CD4+ cell count over time among hiv seropositive gay men. Psychology & Health. 2004;19(2):183-96.

22. Horowitz M, Sonneborn D, Sugahara C, Maercker A. Self-regard: a new measure. The American journal of psychiatry. 1996;153(3):382-5.

23. Cheek JM, Briggs SR. Aspects of Identity Questionnaire (AIQ-IV) . . Measurement Instrument Database for the Social Science. 2013.

24. Aunger R, Curtis V. Behaviour Centred Design: Toward and applied science of behaviour change. . Health Psychology Review 2016.

25. D'Mello-Guyett L. 7th Emergency Environmental Health Forum (EEHF) Event Report. UK: SHARE consortium; 2016.

26. George CM, Biswas S, Jung D, Perin J, Parvin T, Monira S, et al. Psychosocial Factors Mediating the Effect of the CHoBI7 Intervention on Handwashing With Soap. Health Education & Behavior. 2017:1090198116683141.

27. Vujcic J, Ram PK, Blum LS. Handwashing promotion in humanitarian emergencies: strategies and challenges according to experts. Journal of Water Sanitation and Hygiene for Development. 2015;5(4):574-85.

28. Aunger R, White S, de Witt Huberts J, Greenland K. Behaviour Centred Design: Formative Research Tools. UK: London School of Hygiene and Tropical Medicine. ; 2017.

29. Greenland K, White S, Sommers K, Biran A, Burton MJ, Sarah V, et al. Selecting behaviour change priorities for trachoma ‘F’ and ‘E’ interventions: A formative research study in Oromia, Ethiopia. PLOS Neglected Tropical Diseases. 2019;13(10):e0007784.
