# Supplementary material for: How does handwashing behaviour change in response to a cholera outbreak? A qualitative case study in the Democratic Republic of the Congo
Source: PLoS One. 2022 Apr 12;17(4):e0266849. doi: 10.1371/journal.pone.0266849 (PMC9004767; doi:10.1371/journal.pone.0266849)
Supplement: S5 Table — (DOCX) [file pone.0266849.s005.docx]

Supplementary Materials - 4

Table 1: Identified determinants and their associated influence on handwashing behaviour in Eastern DRC.

| **Determinants** | | **Type of effect on handwashing behaviour** |
| --- | --- | --- |
| **Individual characteristics** | Gender (being female) | Positive |
|  | Age (being a child or an older person) | Negative |
|  | Personality (Being a person who values cleanliness) | Positive |
|  | Facing extreme poverty | Negative |
|  | Having chronic physical health challenges | Negative |
|  | Permanent or regular employment | Positive |
|  | Ethnicity | None |
|  | Religion | None |
|  | High level of education | None |
|  | Being a single person household | Negative |
|  | Having mental health challenges | Negative |
| **Capabilities** | Perceived inability to afford soap | Negative |
| **Physical environment** | Exposure to dust or mud | Positive |
|  | Living in a rural area | Mixed |
|  | Dry season | Negative |
|  | Living environments that are perceived to be dirty and hard to clean | Negative |
| **Social Environment** | Family members who encourage handwashing | Positive |
|  | Frequent reminders from NGOs about handwashing | Positive |
|  | Ability to borrow soap and water from others | None |
|  | Sociality and interaction with others | None |
|  | No social judgement or social sanctions if handwashing is not practiced | Negative |
| **Stage** | Limited space within households | Negative |
|  | Using shared WASH facilities | Negative |
| **Infrastructure** | Having insufficient access to water (due to costs or inconsistent supply) | Negative |
|  | Having to walk a long distance to fetch water | Negative |
|  | Having insufficient jerry cans to collect and store water | Negative |
|  | Having a dedicated handwashing facility | Positive |
|  | Using grey water for handwashing | Positive |
| **Props** | Having insufficient access to soap | Negative |
|  | Soap not kept in a convenient location | Negative |
|  | Having access to ash | None |
| **Roles** | Being an IDP | Negative |
| **Routine** | Majority of time spent outside of the house for work | Negative |
|  | Unpredictability of circumstances | Negative |
|  | Frequency of other household tasks involving soap and water | Positive |
| **Norms** | Handwashing is seen as something that is socially approved | None |
|  | Perceived frequency of handwashing practices of neighbours, friends and family | None |
|  | Belief that more people are practicing handwashing during the cholera outbreak | Positive |
| **Executive Brain** (including knowledge, beliefs and risk) | Knowledge about the role of handwashing in interrupting disease transmission | Mixed |
|  | Knowledge of key times for handwashing | None |
|  | Perceived effectiveness of handwashing in preventing cholera | Positive |
|  | Belief that some exposure to dirt is healthy | Negative |
|  | High perceived severity of cholera | Positive |
|  | Low perceived vulnerability to cholera (including due to belief that it would affect Congolese people) | Negative |
| **Discounts** | Prioritisation of soap and water for other tasks | Negative |
|  | Busyness and tiredness | Negative |
|  | Concern about other problems | Negative |
| **Reactive Brain** | Absence of cues to trigger handwashing at key times | Negative |
| **Motivated Brain** | Fear | Positive |
|  | Hunger | Negative |
|  | Nurture | Mixed |
|  | Status | Positive |
|  | Comfort | None |
|  | Hoard | Negative |
|  | Attract | Positive |
|  | Love | Positive |
|  | Affiliation | None |
|  | Disgust | Positive |
