# Supplementary material for: How does handwashing behaviour change in response to a cholera outbreak? A qualitative case study in the Democratic Republic of the Congo
Source: PLoS One. 2022 Apr 12;17(4):e0266849. doi: 10.1371/journal.pone.0266849 (PMC9004767; doi:10.1371/journal.pone.0266849)
Supplement: Attachment — Submitted filename: review of DRC handwashing mansucript.docx [file pone.0266849.s006.docx]

Referee review for: "How does handwashing behavior change in response to a cholera outbreak? A qualitative case study in the Democratic Republic of Congo”

This study uses a mixed methods approach to examine perceptions and behaviors associated with handwashing with soap among households that had experienced cholera and households that had not experienced cholera during a cholera outbreak. The outbreak took place in 2017 in a resource poor community in the eastern DR Congo where endemic and epidemic cholera exists. Health education messages involving biomedical information about the health benefits of handwashing had been disseminated over time in the study community. Study results suggest the handwashing with soap was rare during the outbreak among both case and control households despite self-reports of handwashing. The findings highlight that competing demands related to food consumption and livelihoods constrained people living in both case and comparison households from practicing handwashing. The authors conclude that severe psychological, social, and economic impacts that occurred in case households increased barriers to handwashing. The authors underline that handwashing strategies in contexts with endemic and outbreaks of cholera should consider emic understandings of disease and promote sustainable strategies that take into account social and physical environmental factors, including access to handwashing materials, that influence handwashing behavior.

Comments

1. Little information is provided on the cholera outbreak. It would be helpful to include a description of the outbreak including how long it lasted and the number of cases and deaths.
2. The manuscript would benefit from a description of the messages provided to community members, including how messages were disseminated, the content of messages and how this changed during the epidemic, the frequency of dissemination, and the target audience. Were the messages focused on diarrhea prevention or were cholera-specific messages disseminated?
3. It is not clear how focus group discussion participants were selected. Also, a description of the composition of the focus groups is needed.
4. While the authors mention that some households participated in more than one method, there is no indication regarding the extent to which IDIs were carried out in households where structured observations took place. The methods section should provide a description indicating when more than one method was carried out in one household.
5. Little is said about the differences between the IDP and local populations and how their living environments and daily routines affected exposure to interventions, perceptions of cholera, and handwashing practices. It would be helpful to include a description of the different social and physical environments, as well as exposure to messaging, to understand how this may have improved or constrained handwashing. Also, a description of differences in the way the outbreak impacted IDPs and the local population would be helpful.
6. The study results suggest the cholera is a general term used to describe a range of diarrheal illness. However, there is no mention of how this may have influenced emic views and the cholera explanatory disease model related to disease severity, causal explanations, and transmission, as well as the perceived need to wash hands. In other words, to what extent were informants able to distinguish disease signs, symptoms, severity, causation, and transmission from other types of diarrheal illness.
7. The authors rightly advocate for incorporating emic views and local constructions of disease into messaging and intervention strategies. However, the manuscript lacks some key information on the local explanatory disease models that could potentially be useful when informing strategies. I wonder whether some of this information was collected during the participatory methods that were not presented in the results. If so, it would be useful to include more details on the local explanatory model of cholera and how this may have affected handwashing behaviors. In that regard, what were the results of the participatory methods and how were the findings used.
8. In the discussion, the authors cite literature suggesting that behaviors may change over the course of an outbreak. It would be helpful to know whether this research detected any behavioral changes over time as perceptions related to the disease and risk of exposure may have changed.

Other comments

Recent research carried out in an IDP camp in North Kivu, which highlights similar findings related to the prioritization of competing needs over handwashing, should be mentioned.

Some statements made in the first paragraph of the discussion section do not necessarily reflect the study findings. Examples include:

- Cholera is conceptualized as a health challenge that is easily treatable
- Handwashing was hampered due to the fact that the work took place in a conflict zone
- Health promotion has led to high levels of knowledge related to the health impacts of cholera
