# Supplementary material for: How does handwashing behaviour change in response to a cholera outbreak? A qualitative case study in the Democratic Republic of the Congo
Source: PLoS One. 2022 Apr 12;17(4):e0266849. doi: 10.1371/journal.pone.0266849 (PMC9004767; doi:10.1371/journal.pone.0266849)
Supplement: Attachment — Submitted filename: Responses to reviewers.docx [file pone.0266849.s007.docx]

**Journal Requirements:**

1. When submitting your revision, we need you to address these additional requirements.Please ensure that your manuscript meets PLOS ONE's style requirements, including those for file naming. The PLOS ONE style templates can be found at 
[https://journals.plos.org/plosone/s/file?id=wjVg/PLOSOne_formatting_sample_main_body.pdf](https://protect-eu.mimecast.com/s/dit3Cl714TXyLKnhy7DmG?domain=journals.plos.org) and 
[https://journals.plos.org/plosone/s/file?id=ba62/PLOSOne_formatting_sample_title_authors_affiliations.pdf](https://protect-eu.mimecast.com/s/jrLgCmy54uWZvYJu9snpL?domain=journals.plos.org)

*Response: We have reviewed these guidelines and made edits accordingly so that the submission now conforms to all guidelines.*

2.  Please include a complete copy of PLOS’ questionnaire on inclusivity in global research in your revised manuscript. Our policy for research in this area aims to improve transparency in the reporting of research performed outside of researchers’ own country or community. The policy applies to researchers who have travelled to a different country to conduct research, research with Indigenous populations or their lands, and research on cultural artefacts. The questionnaire can also be requested at the journal’s discretion for any other submissions, even if these conditions are not met.  Please find more information on the policy and a link to download a blank copy of the questionnaire here: [https://journals.plos.org/plosone/s/best-practices-in-research-reporting](https://protect-eu.mimecast.com/s/75EVCnO14cmznLOsmcn8g?domain=journals.plos.org). Please upload a completed version of your questionnaire as Supporting Information when you resubmit your manuscript.

*Response: Thanks for flagging this questionnaire. We feel it is a helpful initiative and a move towards decolonising research and increasing transparency. We have completed the questionnaire and have included this as supplementary material and referenced it in text on page 8.*

3. We note that you have stated that you will provide repository information for your data at acceptance. Should your manuscript be accepted for publication, we will hold it until you provide the relevant accession numbers or DOIs necessary to access your data. If you wish to make changes to your Data Availability statement, please describe these changes in your cover letter and we will update your Data Availability statement to reflect the information you provide.

*Response: No changes to make. This information remains correct.*

4. Please amend your authorship list in your manuscript file to include author  Sian White, Anna C Mutula, Modeste M Buroko, Thomas Heath, François K Mazimwe, Karl Blanchet, Val Curtis, Robert Dreibelbis.

*Response: These names were already included in the manuscript so nothing has been changed.*

5. We note you have included a table to which you do not refer in the text of your manuscript. Please ensure that you refer to Table 1 in your text; if accepted, production will need this reference to link the reader to the Table.

*Response: Thanks for identifying this oversight. This is now included in text on page 8.*

6. Please review your reference list to ensure that it is complete and correct. If you have cited papers that have been retracted, please include the rationale for doing so in the manuscript text, or remove these references and replace them with relevant current references. Any changes to the reference list should be mentioned in the rebuttal letter that accompanies your revised manuscript. If you need to cite a retracted article, indicate the article’s retracted status in the References list and also include a citation and full reference for the retraction notice.

*Response: We are not sure if this comment relates to a particular reference. To the best of our knowledge none of these references have been retracted.*

**Reviewer 1 Comments:**

7. Methods: Line 40-42 states the objective was to find out how the people responded to the cholera outbreak and how the outbreak affected their handwashing behavior. Is it right to assume that you wanted to find out if they washed their hands more often after the outbreak? How was this done/measured? was there a measurement of handwashing behavior before the outbreak to compare with that after the outbreak?

*Response: No this is an incorrect assumption. The objective is broader than what reviewer 1 assumes. Given that this was an exploratory study we did not start with a priori assumptions about how behaviour may change during an outbreak.* *The study focuses primarily on the determinants of behaviour (rather than behaviour itself) and how these may have been shifted by the outbreak. While observational measures of handwashing are included, they were only on a small sample and were designed to be qualitatively analysed rather than to indicate handwashing prevalence or frequency. Similarly participants self-reported their behaviour during many of the methods but we know that self-reported handwashing behaviour tends to overestimate practice. We have not adjusted the objective but rather have added some text in the limitations to better explain this.*

8. Results: From table 1, 21% lived in camps while the rest were lived in the community, was there a difference in water supply between the camps and community? Did this difference have any impact on the handwashing behavior?

*Response: This information has been added to the text. In summary there were no substantial differences.*

9: Summary and general comments: Thank you for the privilege to review this well written paper. The authors carried out a matched case-control study to examine the hygienic behavior of the people of Kivu during the 2017 cholera outbreak. These results will be very useful for the cholera outbreak literature. I suggest revising for key elements in the methods and results which need a bit more clarification. Again I commend the authors for clarity of thoughts which allowed me to only focus on the science.

*Response: We thank Reviewer 1 for their kind words of encouragement.*

**Reviewer 2 Comments**

10. Little information is provided on the cholera outbreak. It would be helpful to include a description of the outbreak including how long it lasted and the number of cases and deaths.

*Response: While in DRC data on cases and deaths were shared with us by individual health centers, humanitarian organizations and the district government. However this data was inconsistent and hard to verify. Total case numbers and mortality from the national outbreak are available and have been included in the text but unfortunately due to these data issues we have not been able to include anything more specific for the region. ‘Outbreaks’ of cholera in DRC are, sadly unending, with this region reporting endemic cases every week of the year. Therefore and outbreak within the DRC context refers to a dramatic rise in cases as is typically experienced most years between about August and January. We have not given this detail in the text as we have already mentioned that our study took place during the peak of the outbreak during October and November.*

11. The manuscript would benefit from a description of the messages provided to community members, including how messages were disseminated, the content of messages and how this changed during the epidemic, the frequency of dissemination, and the target audience. Were the messages focused on diarrhea prevention or were cholera-specific messages disseminated?

*Response: This was not assessed formally as part of this study. We have provided some additional description of this based on what participants told us. However we did conduct some parallel research with humanitarians about their programming throughout the Eastern region of DRC. This work has been submitted separately to Conflict and Health.*

12. It is not clear how focus group discussion participants were selected. Also, a description of the composition of the focus groups is needed.

*Response: Information on sampling is provided under the heading ‘sampling’ in the methods section. We have expanded on this information about the composition of the FGDs under this section of the methods.*

13. While the authors mention that some households participated in more than one method, there is no indication regarding the extent to which IDIs were carried out in households where structured observations took place. The methods section should provide a description indicating when more than one method was carried out in one household.

*Response: We have added this information under the IDI section.*

14. Little is said about the differences between the IDP and local populations and how their living environments and daily routines affected exposure to interventions, perceptions of cholera, and handwashing practices. It would be helpful to include a description of the different social and physical environments, as well as exposure to messaging, to understand how this may have improved or constrained handwashing. Also, a description of differences in the way the outbreak impacted IDPs and the local population would be helpful.

*Response: As noted above we have added some information on the similarity of WASH circumstances between IDPs and host communities. In the text we had tried to describe the different housing of both populations. Since these camps were informal and located within the heart of the communities there was little difference in exposure to hygiene promotion, however those outside the center of town were slightly less exposed as is now noted in the text. We have also included more detail on employment.*

15. The study results suggest the cholera is a general term used to describe a range of diarrheal illness. However, there is no mention of how this may have influenced emic views and the cholera explanatory disease model related to disease severity, causal explanations, and transmission, as well as the perceived need to wash hands. In other words, to what extent were informants able to distinguish disease signs, symptoms, severity, causation, and transmission from other types of diarrheal illness.

*Response: We have added content to the limitations section of the paper to address this point. We agree that this may have biased or distorted their reporting of experiences and perceptions towards the disease, however we don’t feel that this would have had a substantial bearing on understandings of disease causation and transmission given that this would be similar to many other diarrheal diseases (at least at a lay population level). Under the knowledge heading in the text I have added that while people viewed it as a catch all term people were able to easily identify cholera-specific symptoms such as rice-water stools.*

16. The authors rightly advocate for incorporating emic views and local constructions of disease into messaging and intervention strategies. However, the manuscript lacks some key information on the local explanatory disease models that could potentially be useful when informing strategies. I wonder whether some of this information was collected during the participatory methods that were not presented in the results. If so, it would be useful to include more details on the local explanatory model of cholera and how this may have affected handwashing behaviors. In that regard, what were the results of the participatory methods and how were the findings used.

*Response: As Reviewer 2 suspected the research aims and methods did not specifically seek to comprehensively understand local explanatory disease models. However quite a lot was learned about this during some of the participatory methods used in the IDIs and FGDs (e.g. personal histories, risk scaling, and cholera case descriptions). Most of these findings we have tried to capture in our results already. Specifically the following points are linked to this:*

- *Cholera is seen as a challenge closely associated with poverty and hunger*
- *Cholera is associated with people who are thin, pale and sickly (ie these individuals are considered more vulnerable).*
- *Understandings of cholera were aligned with western biomedical models of understanding disease (this point has been added in text) given the history of health promotion in the region.*
- *A minority of people felt that Congolese people are resilient to cholera or that if they got infected it wouldn’t cause them serious illness.*
- *A minority of people felt that if you are too clean you would actually be more vulnerable to cholera.*
- *A minority of people felt that it was in humanitarian and government interests for cholera outbreaks to continue.*
- *People who had had cholera tended to be more likely to see cholera as being random.*

*In terms of the second part of your question, participatory methods were used in throughout each of the IDIs and FGDs (see supplementary materials for full descriptions). Therefore all the content included in the results relates to these methods.*

17. In the discussion, the authors cite literature suggesting that behaviors may change over the course of an outbreak. It would be helpful to know whether this research detected any behavioral changes over time as perceptions related to the disease and risk of exposure may have changed.

*Response: It is perhaps worth noting that the literature cited in relation to this point is limited by its quality. As noted in text that is because the majority of these studies rely on self-reported behaviour. As noted in the text the consensus across such texts is that behaviour seems to fluctuate over the course of many outbreaks and that one of the many factors driving this appears to be perceptions about the disease and the fear associated with it. This indeed is the premise behind Terror Management Theory which is referenced in the text. Given that we feel we have already explained this literature and its implications we have not added anything to this section.*

18. Recent research carried out in an IDP camp in North Kivu, which highlights similar findings related to the prioritization of competing needs over handwashing, should be mentioned.

*Response: We have added in some additional content into the discussion section based on the work of Blum et al 2019 and Claude et al 2020.*

19. Some statements made in the first paragraph of the discussion section do not necessarily reflect the study findings. Examples include: a) Cholera is conceptualized as a health challenge that is easily treatable b) Handwashing was hampered due to the fact that the work took place in a conflict zone, c) Health promotion has led to high levels of knowledge related to the health impacts of cholera.

*Response: In relation to point a) this was mentioned in our results but has now been amended so this is a bit more explicit. In relation to point b) our discussion doesn’t quite claim that handwashing is hampered because of the conflict but rather the social and environmental factors that are associated with poverty, and the circumstances crated by conflict and displacement. We have tried to further clarify this nuance in the text. In relation to point c) We realize that while mentioned the high levels of knowledge in the results section we left out information on where participants felt this knowledge came from. This has now been added to the results section so this link is clearer.*
